# Review of Magnesium Wheel Types and Methods of Their Manufacture

**DOI:** 10.3390/ma17030584

**Published:** 2024-01-25

**Authors:** Anna Dziubinska, Ewa Siemionek, Piotr Surdacki, Monika Kulisz, Bartosz Koczurkiewicz

**Affiliations:** 1 Metal Forming and Casting Department, Faculty of Mechanical and Industrial Engineering, Warsaw University of Technology, Pl. Politechniki 1, 00-661 Warsaw, Poland; 2 Department of Automotive Vehicles, Faculty of Mechanical Engineering, Lublin University of Technology, Nadbystrzycka 38D, 20-618 Lublin, Poland; e.siemionek@pollub.pl; 3 Department of Metal Forming Technologies, Faculty of Mechanical Engineering, Lublin University of Technology, Nadbystrzycka 38D, 20-618 Lublin, Poland; piotr.surdacki@pollub.pl; 4 Department of Organisation of Enterprise, Faculty of Management, Lublin University of Technology, Nadbystrzycka 38D, 20-618 Lublin, Poland; m.kulisz@pollub.pl; 5 Department of Materials Science and Engineering, Faculty of Production Engineering and Materials Technology, Czestochowa University of Technology, 69 J.H. Dąbrowskiego, 42-201 Czestochowa, Poland; bartosz.koczurkiewicz@pcz.pl

**Keywords:** magnesium alloy, wheels, type of wheels, wheel-manufacturing methods, casting, metal forming

## Abstract

This article provides a detailed review of the types of magnesium wheels available in the industry and the current methods of the wheels’ production. The past several years have seen a significant development of magnesium-based lightweight alloys employed as a structural material for modern light vehicles. Magnesium alloys are characterized by their low density while maintaining good mechanical properties. The use of these alloys in the industry enables vehicles’ weight reduction while increasing their technical parameters. The first part of the article presents the unique properties of magnesium alloys that determine the application of this material for lightweight vehicle wheels. The advantages of using magnesium wheels over aluminum wheels are also presented. Next, a classification of the types of magnesium wheels was made in regard to their construction, applications, and manufacturing methods. At present, magnesium wheels by construction can be classified according to their geometry as single parts or assembled parts. In reference to geometry, wheels can have different shapes: classic, multi-spoke, with holes, or with frames. Depending on the geometry used, magnesium wheels can have different parameters, such as their mounting hole spacing, wheel diameters, or rim width. Considering the applications in various industries, main distinctions can be made between magnesium wheels for automobiles, motorcycles, bicycles, and wheelchairs. Magnesium wheels can also be categorized in regards to the manufacturing methods: casting, machining, forging, and hybrid manufacturing. The second part of the article focuses on the analysis of magnesium alloy wheel-manufacturing technologies used in the industry and developed by research centers. This article discusses these manufacturing technologies in detail and indicates prospective directions for further development.

## 1. Introduction

**Figure 1:**
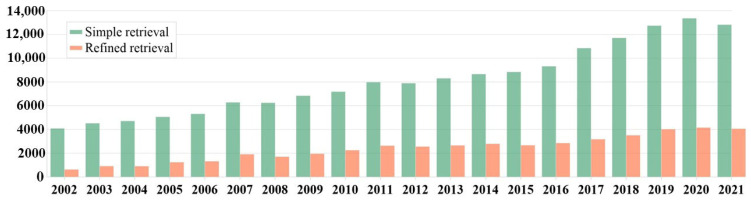
Articles focused on magnesium alloys published in the Web of Science database in the past 20 years (own study based on the literature [1]).

The use of lightweight metals as construction materials is regarded a crucial importance. Over the past few years, a significant development in lightweight magnesium-based alloys occurred. Additionally, the interest of scientific centers in research concerning magnesium alloys spiked as well [1] (Figure 1).

There is a growing interest among automotive manufacturers in producing magnesium alloy components. It is expected that magnesium consumption in a modern vehicle may soon exceed 100 kg^2^ [2,3]. Magnesium is the lightest structural material available today [1,4]. It is 1.5 times less dense than aluminum and 4.5 times less dense than steel. Therefore, magnesium wheels can be designed to be much lighter than aluminum wheels while exhibiting comparable strength properties. Table 1 shows a comparison of magnesium wheels with aluminum wheels and steel wheels. 

**Figure 2:**
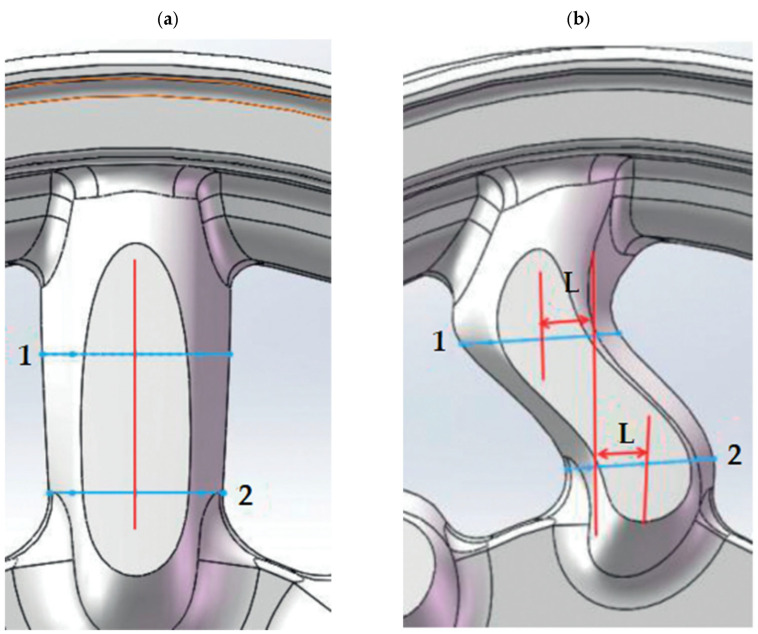
Offset positions due to structural optimization: positions 1 and 2 shifted left and right along the center, L—the size of the adjustments according to the Table 2 [12].

**Figure 3:**
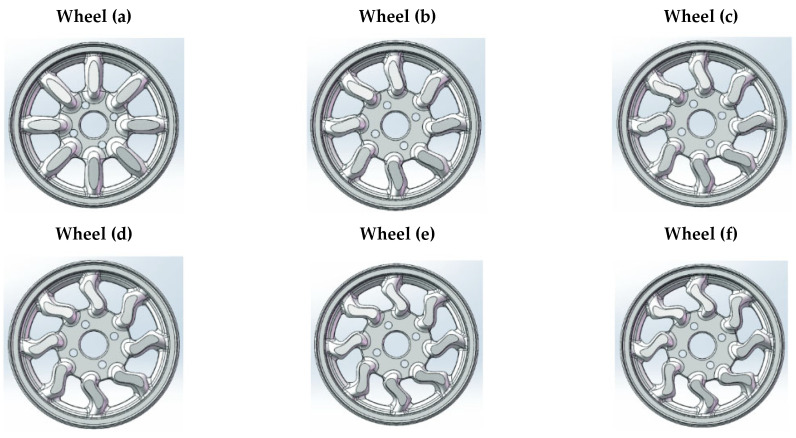
Different wheel structures obtained after optimization with the size of the adjustment L according to Table 2 [12].

An example of magnesium alloy applications is wheels in racing vehicles. Forged monoblock lightweight alloy wheels combine the lightweight construction of special magnesium alloys with superior stability. The lightweight design of the wheels significantly reduces the volume of rotating and unsprung masses while greatly increasing the stability and load-bearing capacity of the wheels. This significantly reduces weight—in some cases by as much as several kilograms per wheel. At the same time, resistance to flange deformation increases by up to 60%. Formula 1, DTM, Indy, NASCAR, and MotoGP wheels are usually made of forged magnesium alloys due to their low density and high strength [7,8]. They are made from a single piece of material (monoblock) to ensure their strength is as strong as possible. They are attached to suspension uprights with a single central wheel mounting nut. Formula 1 wheels must be made of AZ70 or AZ80 magnesium alloys. However, regulations are not as restrictive in other racing classes. An application example is the use of magnesium-cast wheels for the Lotus F1 race cars of 1958 [7,9]. Typically, the ZK60 alloy is used for wheels used in car or motorcycle racing wheels [9]. The G-AlSi7Mg and AZ91 alloys are used for monocoque wheels with a diameter of 17″ to 22″, while the G-AlSi7Mg is used for mounted wheels from 18″ to 23″ [10]. Wheels are of critical importance for vehicles because they not only carry the weight of the entire vehicle, but the rotating tire also transmits horizontal force and impact force from the road surface. Jiang and Lan’s research designed a lightweight AZ91 alloy vehicle wheel [11]. The wheel model is lightweight while ensuring safety. The same alloy was used by Jiang et al. in their research [12], in which they optimized the wheel design to meet lightweight and dynamic impact resistance requirements by optimizing the topology of the wheel structure. They changed the wheel spoke structure in combination with structural damping characteristics to design different wheel structures (Figure 2 and Figure 3). In addition to the AZ91, aluminum 6061-T6 alloy and SPFH540 steel were used in this research. Testing of the damping properties of the materials revealed favorable damping properties for the magnesium alloy material. Compared with the aluminum alloy wheel, the magnesium alloy wheel design can reduce weight by 32.3%. To optimize the wheel, the spoke design was adjusted (Figure 2 and Figure 3). Based on the wheel optimization (Table 2), material damping, and structural damping, combined with the analysis of stress and total strain, it was found that the most significant structure was that of the wheel shown in Figure 3b. 

Examples of the use of magnesium alloys in the automotive industry can be seen in the VW family car chassis, among others. Potential applications include wheels and components such as control arms, subframes, and brackets. Berlin Transportation Co. uses Mg wheels in its coaches not only to improve handling characteristics but also to extend tire life [13].

Magnesium alloys are also used in wheel hubs. Qi et al., in their research, made a wheel hub from the ZK61-Y magnesium alloy using a liquid-forging process, isothermal forging [14]. They investigated the effect of the Y element content on the microstructure and mechanical properties of liquid-forging blanks. The Mg-1YL alloy showed the best properties with an elongation of 16%, a yield strength of 104 MPa, and a tensile strength of 232 MPa. In addition to wheel hubs, magnesium alloys are used for rims, as presented in the study by Li et al., who investigated the fatigue properties and deformation behavior of rims made from the magnesium alloy Mg-2.96Nd-0.21Zn-0.39Zr [15].

Despite the great interest in magnesium alloys for making magnesium wheels, there are some limitations due to the disadvantages of magnesium alloys. Among the significant disadvantages of magnesium alloys is their flammability. Magnesium wheels have been banned in some forms of motorsports in the UK [6]. Among other disadvantages of magnesium alloys is the low corrosion resistance of some alloy grades. As a result, special anti-corrosion coatings are applied to magnesium wheels [16]. This problem has been solved with new magnesium alloys. Further limitations include the rather high price of magnesium alloys, which is why magnesium wheels are expensive and are mainly used on sports and luxury vehicles.

The examples of the use of magnesium wheels in scientific publications and applications of automotive manufacturers presented above indicate that this research field is interesting and developing. Numerous articles on magnesium wheels have been published in the past 10 years. However, no publication was found that systematized the knowledge of the types of magnesium wheels regarding various aspects of the type of construction, applications, and manufacturing methods. As a consequence, the authors of this study conducted a detailed literature review and systematized knowledge in this area.

## 2. Types of Magnesium Wheels

Magnesium wheels can be classified according to various criteria, such as their construction, shape, design dimensions, type of magnesium alloy used, production methods, and application. The categorization of magnesium wheels, especially in the context of their application, may be of significance both for producers and users because it may aid in the appropriate selection of wheels for specific operational conditions. The selection of Mg wheels, depending on their application, should take into consideration the following aspects: durability and low weight, technical parameters such as diameter, width, offset, design, and aesthetic features, and conformity with industry standards. Several branches of industry test magnesium wheels before they are introduced to the market in order to determine if the product conforms with the applicable standards. Such an approach guarantees that the product meets specific quality standards, which is critical for ensuring safety during exploitation. Several countries worldwide enforce certifications. In the automotive industry, the wheels of vehicles undergo tests associated with approval certification, i.e., certifying if the new vehicle is roadworthy. The approval certification examines whether the vehicle meets standards and technical requirements. Based upon these tests, the approval certificate is issued, which enables the vehicle to be legally registered. Depending on the country and enforced standards and directives, wheels undergo, inter alia, the following: rolling test, rotating bending test, biaxial fatigue test of the wheel/hub, salt spray chamber test, and corrosion-resistance test. There are a variety of magnesium wheels and rim designs and configurations that must have sufficient strength to effectively support the vehicle and counteract the forces generated during normal operation. A key aspect is to ensure that the wheel design is as lightweight as possible to reduce the overall weight of the vehicle while achieving optimal vehicle performance [17].

Magnesium wheels manifest increased efficiency due to their lower weight. Weight reduction is particularly effective because the wheels, being de-sprung, rotate, generating an effect that, along with tires, takes on an exponential nature compared to other vehicle components. The resulting savings in fuel consumption can reach up to 8% in urban conditions. Lighter wheels turn faster and can brake faster, which contributes to more efficient deceleration of the vehicle under certain conditions [18]. This, in turn, improves braking dynamics, with benefits in terms of increased safety. Wheels with lower weight have a positive effect on the handling and maneuverability characteristics of the vehicle, especially when cornering, even at higher speeds. Magnesium alloys effectively dissipate energy, resulting in lower temperatures in brake systems and hubs [19]. This, in turn, contributes to the longer life of brake pads and adjacent components. Wheels made of magnesium alloy perfectly absorb and dissipate shocks and vibrations. The unique damping properties of magnesium exceed those of aluminum by up to 50 times. Such a mechanism significantly reduces the vibration load on the vehicle, especially in elements such as the engine, suspension, and transmission. This effect contributes to improving the overall performance of the vehicle and extends its life [20].

### 2.1. Types of Magnesium Wheels by Construction

**Figure 4:**
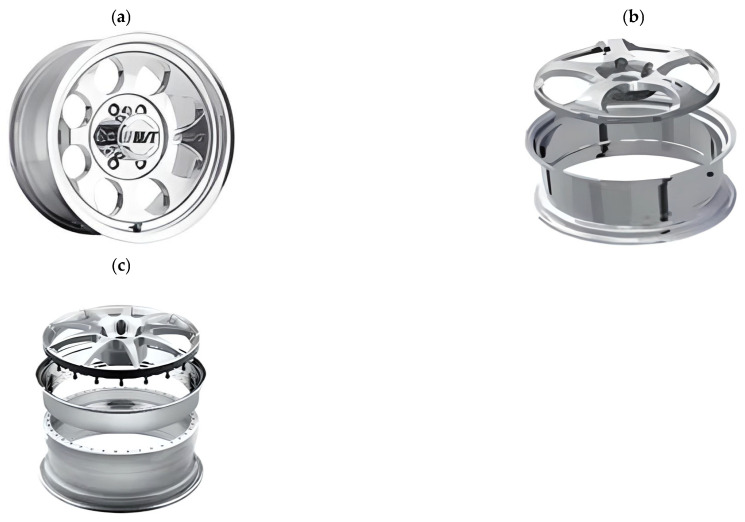
Side view of a single-piece wheel (**a**), two-piece wheel (**b**), and three-piece wheel (**c**) [22].

When construction variants are considered, magnesium wheels can be divided into:

1. One-piece wheels: In this design, the wheel disk and rim are made from a single piece of material. Forged lightweight alloy wheels and cast wheels are dominant in this category (Figure 4);
2. Multi-piece wheels are available mainly for tuning applications and for sports vehicles. Multi-piece wheels originated from motorsports. Mechanics exploited the advantage of being able to replace damaged parts. Multi-piece wheels are divided into two-, three- and five-piece wheels [21]. Three-piece wheels allow for easier replacement of damaged parts and customization of the wheel’s size. Five-piece wheels are the most complex and expensive wheels but offer high quality and durability (Figure 4).

Depending on the geometry used, magnesium wheels have a variety of dimensions and mounting parameters, which can include:

1. Mounting hole spacing determines the distance between the centers of the holes into which the bolts attaching the wheel to the hub are screwed. This parameter is crucial, as it must match the spacing of the holes in the vehicle’s hubs. Failure to match will render mounting the wheel impossible. The spacing of mounting holes in magnesium wheels can vary depending on the specific rim model and vehicle. The predominant mounting hole spacings are as follows: 4 × 100 mm—a common mounting hole spacing, popular especially on older compact and sports cars; 5 × 100 mm—used in many car models, especially in mid-range and sports cars; 5 × 112 mm—this mounting hole spacing is mainly seen in German cars and some Japanese cars; 5 × 120 mm—this mounting hole spacing, is, among others, in BMW cars, but also some cars of other brands; 6 × 139.7 mm—this mounting hole spacing is used in off-road cars and pickups, such as the Toyota Hilux, Mitsubishi L200, Nissan Navara, or Chevrolet Silverado.
2. Wheel diameter is a feature that determines the size of the wheel itself, measured by the outer edge of the rim. This parameter is usually expressed in inches or millimeters, taking different values depending on the car model and the owner’s preferences. Popular wheel diameters, especially in city and compact cars, are 15 and 16 inches. In contrast, 17- and 18-inch sizes are increasingly used on mid-range and high-end vehicles, as well as sports cars and some off-road models and SUVs.
3. Rim width is the dimension that determines the width of the rim itself, measured from the outer to the inner edge of the rim. This parameter has a significant impact on the tire’s traction; the wider the rim, the greater the tire’s contact with the road, which can improve the vehicle’s grip but, at the same time, increase tire wear. The most common magnesium rim widths are 6.5 inches, which are commonly used on many cars, especially city and compact vehicles. Rim widths of 7.0 inches are a popular rim width, especially on mid-range and sports cars, while 7.5 inches is increasingly used on mid- and high-end cars, as well as on some off-road vehicles and SUVs. Rim widths of 8.0 inches and larger are frequently used on sports cars, luxury cars, as well as some off-road models and SUVs.
4. Offset (ET) is the distance between the center of the rim and the plane of attachment to the hub. This parameter determines how far the rim will protrude beyond the car’s fender, having a significant impact on driving stability and vehicle aesthetics. Positive offset (mounting face toward the front face of the) is most common on rear-wheel drive cars and some sport and off-road models. Zero offset (mounting face, even with the center line of the wheel) is commonly used on front-wheel drive cars. Negative offset (mounting face toward the rear of the wheel) is mainly used on sports and tuned cars, allowing for a wider track width.

**Figure 5:**
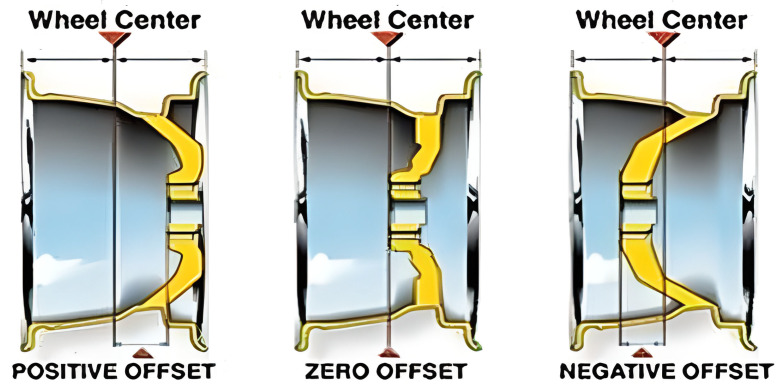
Type of wheel offsets [22].

Choosing the right offset is crucial for maintaining handling characteristics and achieving the desired appearance of the vehicle (Figure 5).

5. The diameter of the centering hole is a parameter that determines the diameter of the inner hole of the rim, which acts as the centering point for the wheel on the hub of the car. It is a key element because proper centering of the wheel on the hub allows for even loading and eliminates vibration while driving. The most common centering hole diameters are as follows: 56.1 mm for front-wheel-drive cars and 66.1 mm for rear-wheel-drive vehicles. On some models, such as the BMW and Mercedes-Benz, the dimension of 72.6 mm is used. For Audi or Volkswagen, this value can also be adjusted. Choosing the right centering hole diameter is crucial for maintaining vehicle stability and comfort [21].
6. Number of mounting holes: This is the number of holes into which the bolts attaching the wheel to the hub are screwed. This number can vary depending on the model of the car and the rim. The numbers 4, 5, or 6 are most typically used.

**Figure 6:**
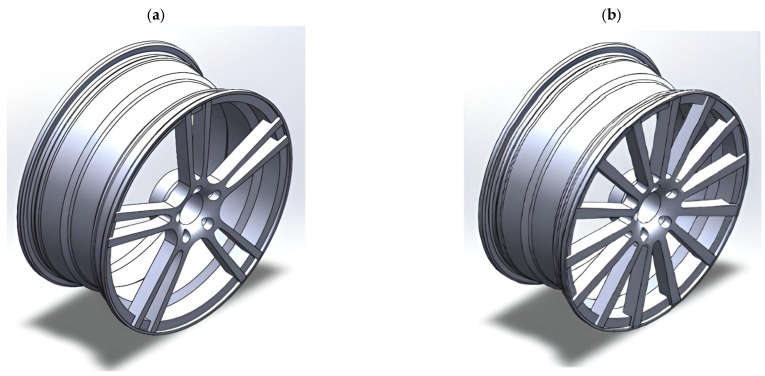
Wheel models used for simulation: (**a**) 5-twin-spoke and (**b**) multi-spoke patterns [23].

Shapes of magnesium wheels can take a variety of forms, depending on the individual preferences of the vehicle’s owner and the prevailing styling trends of the time. Below are some of the most common magnesium wheel shapes:

1. Classic magnesium wheels: Characterized by a simple and elegant design, often used on classic cars and vehicles with simple styling lines;
2. Five-spoke wheels: They have a dynamic, sporty look and are often selected for sports cars and tuned vehicles (Figure 6a);
3. Wheels with multiple spokes: Offer a more complex design, frequently combining sporty and elegant elements. (Figure 6b);
4. Framed wheels: They are characterized by the presence of borders around the edges of the rim, which gives them a unique character and elegance;
5. Holes wheels: They feature cut-out holes along the edge of the rim, which adds to their unique character and sporty style.

**Figure 7:**
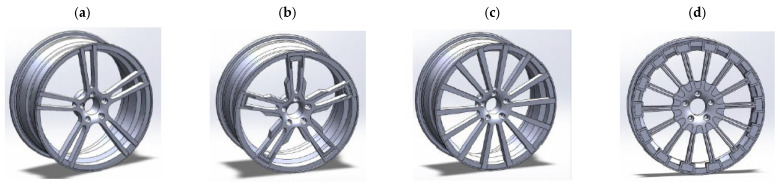
Design of the 5-twin-spoke wheel: (**a**) before and (**b**) after topology optimization, and the multi-spoke wheel (**c**) before and (**d**) after topology optimization [23].

The study of AZ91 magnesium alloy wheels was conducted by Karim and Ku, who analyzed the distribution of stresses and strains in rim designs of various shapes, including 5-spoke and multi-spoke rims made of lightweight materials. In addition, they attempted to optimize the basic designs of the rims to achieve optimal performance levels (Figure 7). The analysis shows that multi-spoke rim designs perform better than 5-spoke ones, both in terms of stress distribution and deformation (Table 3). However, it is worth noting that some weaknesses were identified, particularly in the hub area. In terms of materials, the AZ91D magnesium alloy was found to exhibit a generally higher strain, von Mises stresses, and alternating stresses than the 6061 aluminum alloy [23]. This finding may be important for further research on optimizing magnesium wheel designs, especially in terms of reducing stresses and strains in the hub area, which were identified as a weak point in the analyses. Further research work may focus on improving the design of multi-spoke rims and on the search for new materials with even better mechanical properties for magnesium wheels.

### 2.2. Types of Magnesium Wheels by Production Methods

Depending on the manufacturing method, magnesium wheels can be divided into:

1. Cast Magnesium Wheels: They are produced by pouring molten magnesium or magnesium alloys into a wheel-shaped mold. The alloy is then cooled and hardened, taking its final circular shape. This relatively simple process allows the wheels to be mass-produced, thus making them relatively cheap to manufacture. Although popular on many types of vehicles, they may be less durable than other types of magnesium wheels. Compared to forged wheels, they are heavier and of lower quality. Manufacturing defects that appear in cast wheels include pitting or porosity and a different metallurgical microstructure, such as a larger grain size. In use, cast magnesium wheels have a greater tendency to crack during heavy impact at high speeds, while forged wheels are more prone to bending [24].
2. Forged Magnesium Wheels: They are manufactured by extruding or forging a block of magnesium alloy in high deformation and temperature conditions, which leads to obtaining the desired shape and strength. Forged wheels are valued for their lightness and durability, which makes them preferable on sports and racing cars. The manufacturing process is more complex and expensive than cast wheels, but it offers excellent performance in extreme conditions. This process leads to a more uniform and durable material structure compared to cast wheels. The metal forming of magnesium alloy wheels is a difficult task due to their narrow range of forming temperature parameters and sensitivity to the strain rate [25]. The main phenomenon limiting the manufacture of Mg wheels is cracking, emerging in insufficient or excessive forming temperatures, or at an excessive deformation rate. As a consequence, the plastic-forming process of magnesium wheels is carried out hot on forging machines with low operating speeds while maintaining isothermal temperature conditions during deformation. Magnesium wheels are most frequently forged by plastic deformation in several stages from a bar stock. The delivery of the process takes place in several forging operations, with multiple heats, on multiple tool sets, and with heat treatment between operations. The resulting forgings are then machined (turned on a lathe and milled) into the final wheel shape by removing excess metal from the forged blank. Unlike cast wheels, forged wheels are characterized by a fine-grained structure and better mechanical properties. The process of forging wheels allows a favorable grain pattern to be obtained and the alignment of the directional pattern along the spokes of the wheel to be optimized. Although forged magnesium wheels are 25 percent lighter than cast wheels, they are less commonly used due to their high production cost. Finished magnesium forged wheels must achieve appropriate calibration ratings before they can be marketed. Existing reports show that forged wheels are prone to fatigue cracking at the spokes. During the metal forming of wheel forgings, there is a small deformation at the spokes and a large deformation at the wheel rim. This results in a non-uniform structure, which affects their susceptibility to cracking. Therefore, the topic of increasing deformation in the spoke area is as yet unresolved and is a developmental research direction.
3. Machined Magnesium Wheels: These are manufactured by machining a block of magnesium alloy, including turning, milling, and other mechanical processes that give the wheel its final shape and finish. This approach enables accurate dimensions and high-quality wheel surfaces to be obtained.
4. Hybrid Magnesium Wheels: They combine different production technologies, such as casting, metal forming, and machining, to obtain the desired properties and shape. This approach can combine the advantages of different processes, providing a balanced combination of strength, lightness, and dimensional accuracy [26].

### 2.3. Types of Magnesium Wheels by Application

**Figure 8:**
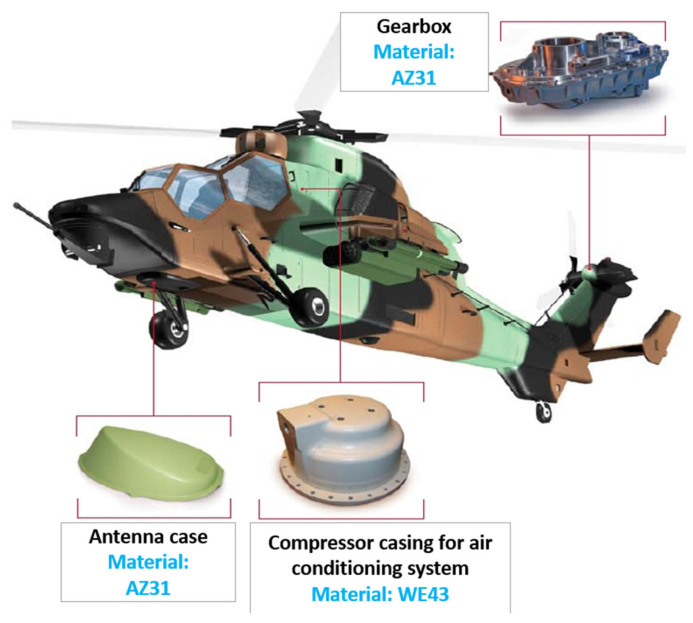
Applications of magnesium alloys in the military industry (own study).

Magnesium wheels are used in a variety of applications, and their functions are valued in many industries. In Śliwa et al. [27] and in the research of Yang et al. [28], the AZ31 material was used to produce wheel hubs employed in aircraft, which reflects the potential of this alloy in the aerospace industry. Research presented in work [27] focused on forming the gear hub by hot-forging from a variety of alloys with different aluminum contents, such as AZ31, AZ61, and AZ80. Even though modern, high-purity magnesium alloys manifest superior corrosion resistance, their use in the aviation industry is limited to engines and transmissions. In modern designs, both in commercial and military aircraft, there is a growing demand for advanced, high-temperature magnesium alloys, such as Elektron^®^, which are characterized not only by their superior parameters but also by corrosion and ignition resistance. Additionally, such alloys meet both efficiency and safety requirements, which may increase the likelihood of their application in constructions requiring high-temperature performance. The application of magnesium is not strictly limited to the aviation industry. Commercial drones, such as DJI Phantom and Mavic, employ magnesium alloys, which form lightweight and durable constructions. For example, the Mavic Air alloy, containing 90% Mg, 9% Al, and 1% Zn, designated AZ91D, is characterized by its low density (1.7 g/cm^3^), which makes it one of the lightest construction metals in the world. These features make the alloy perfect for drone application, offering a balance between durability and mass [29,30]. In the military industry, magnesium-based materials are frequently employed in the components of wheels, radar antennas, radar bases, covers, housing, and missile elements (Figure 8) [31]. The application of magnesium alloys in the automotive industry encompasses not only the production of wheels but also a variety of elements such as steering wheels, engine components, body components, cylinder head covers, seat frames, sunroofs, pedal supports, and many others [17]. 

**Figure 9:**
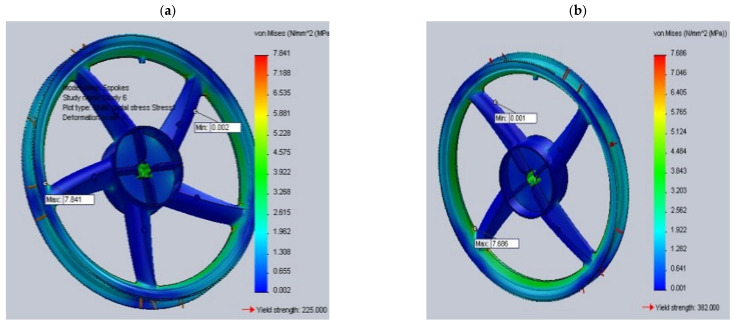
Stress distribution of: (**a**) motorcycle wheel with five spokes and Al alloy; (**b**) motorcycle wheel with four spokes and Mg ZK60 alloy [19].

The largest share of the use of vehicle magnesium wheels is in the automotive industry. They are used in various types of vehicles and are especially popular among motorcyclists. Such wheels are preferred in sports motorcycles due to their lightness. They are frequently used in vehicles, especially when riding on winding roads is predicted to be done frequently. For that reason, they find application in touring motorcycles where reduced mass translates into improved performance and riding comfort. Off-road motorcycles, which are associated with shocks when riding over uneven terrain, often use such wheels. They are frequently used in non-standard motorcycles due to their advantages and the ability to match the style of the vehicle [19]. Due to their low density and superior durability, magnesium wheels are interesting for the producers of sporting and recreational equipment. Magnesium alloys can be employed as structural elements of bicycles, in high-altitude mountaineering equipment, and as structural elements of rollerblades, etc. Integrated magnesium alloy wheels with six or three spokes were introduced in electric mountain bicycles. In such an application, integrated magnesium alloy wheels are lighter than wheels with aluminum alloy spokes. The AM60B alloy is used in electric bicycle applications (Figure 9) [32].

In wheelchairs, magnesium wheels are responsible for the reduced weight of the wheelchair, facilitating movement, most importantly, on uneven terrain. In addition, owing to their stiffness, they enhance handling, which affects the comfort of use by a person with disabilities. Wheelchairs with magnesium wheels are especially appreciated by disabled people who are confined to a wheelchair during such activities as shopping or walking.

Magnesium alloys, e.g., in the form of housing, have found application in various industries. Due to their low weight and capacity to protect against electromagnetic interference, they are employed in the electronics industry as housings of photo cameras, digital recorders, etc. Additionally, magnesium alloys are applied in the electrical machinery industry in the manufacture of power tools due to the alloys’ vibration-damping capacity and in the manufacture of other types of machine components and research equipment. In the construction industry, magnesium alloys are used in the production of windows, window and door handles, bolts, escutcheon plates, decorative elements, and others. In the textile industry, magnesium-based alloys are used in weaving loom bars and in high-speed elements such as coils, spools, and brush holders. The medical industry constitutes a prominent field of application of Mg alloys. Implants, surgical bone staples, as well as surgical sutures are made of the alloys.

## 3. Methods of Manufacturing Magnesium Wheels

Currently, magnesium wheels are produced by the following technologies: casting, machining, and metal-forming processes, including die forging, extrusion, rolling, flow forming, spinning forming, and hybrid forming [7,33,34]. Most of the technologies listed are used by magnesium wheel manufacturers. Among the most popular industrial applications are the two oldest technologies: casting and die forging. The following processes are also used in industrial practice: machining, flow forming, and spinning forming, as well as multi-level hybrid processes. Among the key manufacturers of magnesium wheels are such companies as follows: BBS USA (Braselton, GA, USA), Marvic Wheels (Brunello, Italy), OZ Group (San Martino di Lupari, Italy), Enkei Corporation (Shizuoka, Japan), Marchesini (Curno, Italy), DYMAG (Chippenham, UK), BlueTech Global (Bloomfield Hills, MI, USA), mbDESIGN (Gelnhausen, Germany), iPE (Taiwan, China), RAYS (Osaka, Japan), HRE Performance Wheels (Vista, UK), Tan-ey-sya (Imizu, Japan), SMW (Riga, Lithuania), APP Tech Forged Wheels (Mestrino, Italy), Ronal Group (Härkingen, Switzerland), MKW Alloy (City of Industry, CA, USA), Minilite (Telford, UK), Washi Beam (Nottingham, UK), and Cromodora Wheels (Ghedi, Italy). Casting technologies for manufacturing magnesium wheels are used by the following companies: Marchesini (Curno, Italy), BBS USA (Braselton, GA, USA), DYMAG (Chippenham, UK), Marvic Wheel (Brunello, Italy), Enkei (Shizuoka Prefecture, Japan), and OZ Group (San Martino di Lupari, Italy). On the other hand, die-forging technology is used to form magnesium wheels by the following manufacturers: Blue-Tech Global (Bloomfield Hills, MI, USA), mbDESIGN (Gelnhausen, Germany), BBS USA (Braselton, GA, USA), iPE (Taiwan, China), RAYS (Osaka, Japan), DYMAG (Chippenham, UK), Tan-ey-sya (Imizu, Japan), SMW (Riga, Lithuania), OZ Group (San Martino di Lupari, Italy), and APP Tech Forged Wheels (Mestrino, Italy). The flow-forming process for manufacturing magnesium alloy wheels is used by HRE Performance Wheels (Vista, UK) and BBS USA (Braselton, GA, USA). Spinning flow technology is used by magnesium wheel manufacturer Enkei (Shizuoka, Japan). The machining technique for manufacturing magnesium wheels is used by the companies Marvic Wheel (Brunello, Italy), BBS USA (Braselton, GA, USA), and OZ Group (San Martino di Lupari, Italy). Some of these manufacturers use hybrid processes composed of several forging techniques, e.g., forging, flow forming and machining, or other combinations. 

### 3.1. Casting of Magnesium Wheels

Today, cast magnesium wheels are most frequently used in industrial applications [35,36,37]. This is due to their greater availability on the market associated with well-developed casting technology and lower prices. Foundries specializing in nonferrous metals have the technological equipment and experience to produce cast magnesium wheels on a large scale. Cast magnesium wheels are characterized by high dimensional accuracy and lower manufacturing costs [38,39,40]. The most common types of casting used are gravity casting [41], high-pressure casting (HPC) [41], or low-pressure casting (LPC) [41,42,43,44], which is much more commonly used [15,45,46].

Due to its strong reactivity and the need for a protective atmosphere, magnesium alloys are most often cast in the process of hot or cold chamber die casting. The process yields products with high properties. Die casting offers attractive flexibility in the design and manufacture of lightweight metal components, including wheels. Magnesium die castings can be designed with thin walls in areas where the product is not expected to have high strength and with thicker walls in those with higher strength requirements. Magnesium can be cast with walls as thin as (1–1.5 mm).

**Figure 10:**
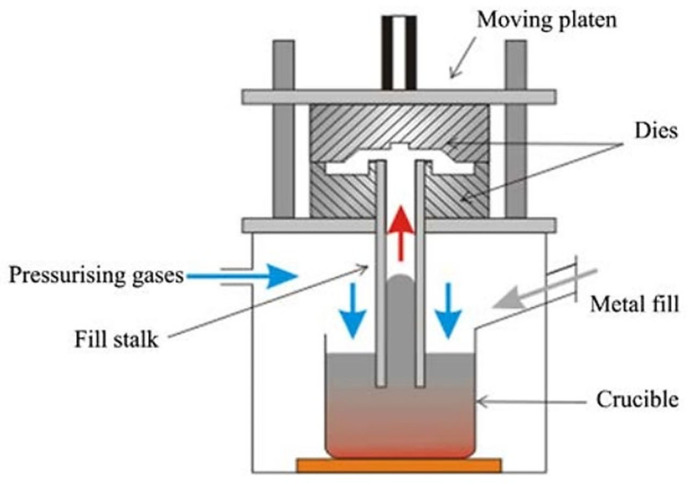
Diagram of a low-pressure casting machine used for casting magnesium wheels [6].

Figure 10 shows a machine used for low-pressure casting of magnesium wheels. It can use sand or metal molds. The LPC machine usually includes a crucible below the mold table with a feed pipe (riser pipe). As shown in the figure, dry gas is used to pressurize the surface of the molten metal in the crucible at a relatively low pressure, which is sufficient to overcome the difference between the die and the surface of the molten metal in the crucible and force the molten metal to rise through the feed pipe, feeder, and filler system into the die cavity [41]. 

Although the low-pressure-casting process involves lower capital expenditures compared to the high-pressure-casting process, the production of components with walls of less than 3 mm of thickness poses a limitation. Additionally, the production cycle takes longer. Table 4 compares the advantages and disadvantages of low-pressure die casting and high-pressure die casting. Hollow castings can be manufactured in the LPC process. On the other hand, such castings cannot be made in the HPDC process. As a consequence, high material savings can be obtained when applying low-pressure casting to produce magnesium wheels. Hollow castings are beneficial due to their structure. Studies concerning the LPC technology for the AZ91 and AM50 magnesium alloys [43,44,45] indicated that LPC castings have superior characteristics when compared with products manufactured by means of gravity casting.

**Figure 11:**
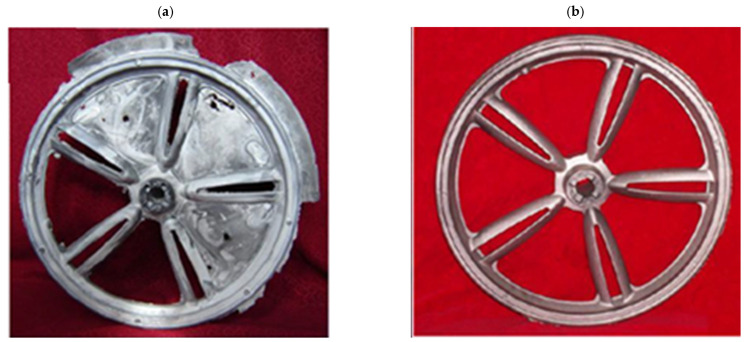
Magnesium wheels obtained by pressure-casting process: (**a**) wheel blank is filled; (**b**) hub surface is smooth [42].

The work of [45] discusses simulations of the low-pressure casting of the magnesium wheel. The simulation of the casting process was conducted for the AZ91D alloy. Simulations of filling, cooling, and solidification were performed by means of casting simulation software. Low-pressure die casting of a magnesium alloy AZ91D wheel was carried out with the runner gate setting position in the center of the wheel for the following parameters: a pouring temperature of 690 °C, an initial mold temperature of 400 °C, and a velocity at the gate of 500 mm/s. Actual motorcycle magnesium wheels, obtained by high-pressure casting technology, are shown in Figure 11 [42]. The following parameters were adopted:

- Pouring temperature in the range of 650–710 °C;
- Injection pressure ratio in the range of 70–110 MPa;
- Mold temperature in the range of 200–300 °C;
- Holding time in the range of 15–30 s.

The objective of the examination was to evaluate the impact of the low-pressure-casting process on the mechanical properties of the formed wheel. Based upon the examination, it was determined that the pouring temperature had the largest impact on the mechanical properties. For the optimal parameters of the casting process, i.e., a pouring temperature = 680 to 700 °C, an injection pressure ratio = 82 to 100 Mpa, a mold temperature = 240 to 280 °C, and a holding time of 20 to 25 s, the following mechanical characteristics were obtained: a tensile strength = 218 to 227 Mpa, an elongation = 9.8 to 10.7%, an impact toughness = 17.5 × 104 to 18.7 × 104 J/m^2^, and a Brinell hardness = 66 to 71 HBS. The test proved that the low-pressure die-casting process is suitable for the manufacturing of magnesium wheels and is fit for mass production.

Currently, gravity casting of magnesium wheels is carried out in sand molds, in dies, or by the full mold and melted model method. Gravity casting of magnesium wheels is rarely used due to inferior mechanical properties of castings, surface roughness, and lower dimensional accuracy compared to wheels obtained by pressure casting [41].

### 3.2. Die Forging of Magnesium Wheels

**Figure 12:**
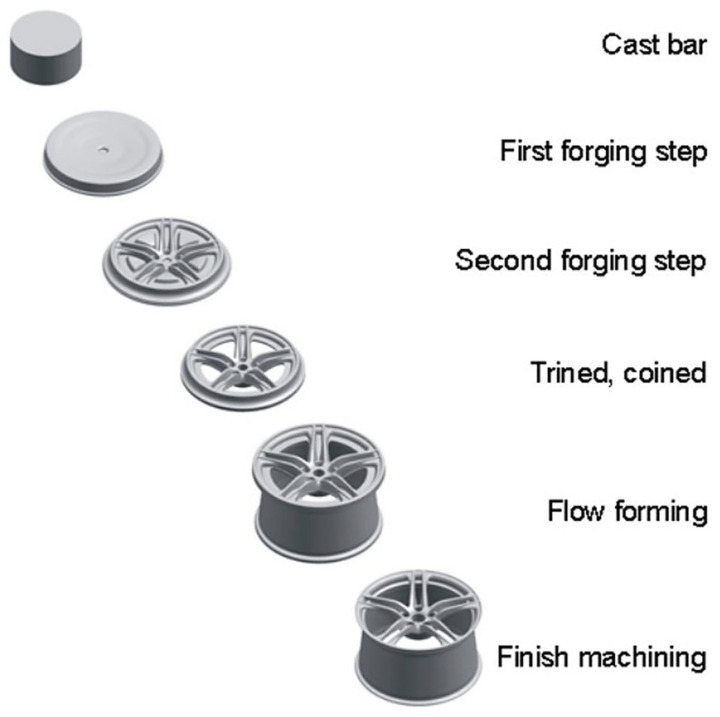
Multi-stage process of forming forged wheels [22].

Die forging is an alternative technology for manufacturing magnesium alloy wheels with high strength requirements [9,33,48,49]. Products with geometry similar to the finished wheel are manufactured [50,51,52]. Magnesium wheels are most often forged by plastic deformation in several stages from a round bar stock (Figure 12). The process takes place in several forging operations with multiple heats on multiple tool sets and with heat treatment between operations [53]. The resulting forgings are then machined (turned on a lathe and milled) into the final wheel shape by removing excess metal from the forged blank.

**Figure 13:**
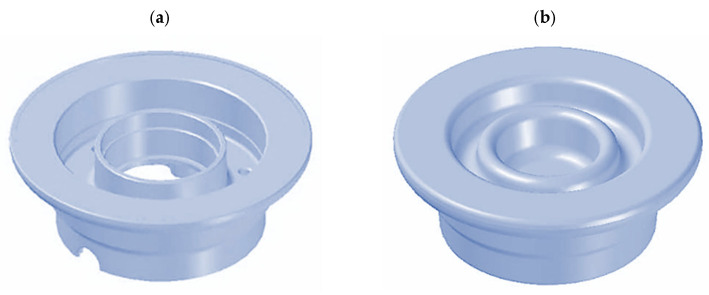

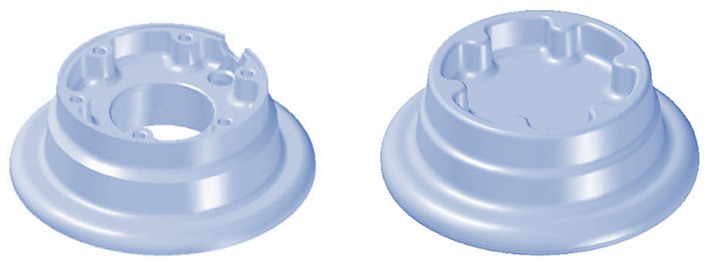
Three-dimensional geometrical model of (**a**) finished magnesium wheel hub and (**b**) forging of the magnesium wheel hub (own study based on the literature [54]).

The work in [54] discusses the results of simulations and experiments conducted in industrial conditions consisting of hammer forging and screw press forging of a small aircraft magnesium wheel hub. Figure 13 outlines the geometrical model of the magnesium wheel hub and its forging. 

The following chief operations are to be completed in the designed technology of die forging of a magnesium wheel hub:

- Cutting billet (round extruded bar) to measure a diameter of 100 mm and height of 93 mm;
- Heating of the billet;
- Initial forging;
- Quality control;
- Heating of the preform;
- Forging in the final impression;
- Cutting of the flash;
- Supersaturation and aging;
- Quality control;
- Assessment of mechanical properties;
- Machining the final product.

**Figure 14:**
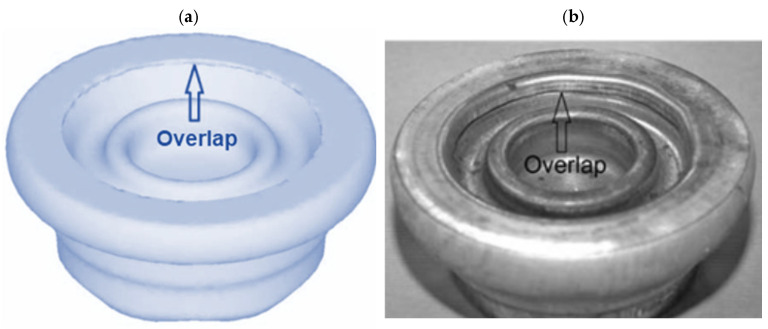
Forging lap in the forging produced with insufficient upsetting of the billet: (**a**) results of simulations and (**b**) results of experimental tests (own study based on the literature [53,54]).

Prior to forging, a theoretical analysis was conducted based on FEM simulations in order to verify the research assumptions. The analysis employed Deform 3D software (v. 11, Scientific FormingTechnologies Corporation, Columbus, OH, USA). An assumption was made that the forging technology of the aviation magnesium wheel hub would be verified in industrial conditions in Zakład Obróbki Plastycznej in Świdnik for the following materials: AZ31, AZ61, AZ80, and WE43. Several billet heating temperatures were scheduled to be tested (Stage 1: 350 °C and 410 °C, Stage 2: temperatures 420 °C and 450 °C). Based on these tests, the most appropriate magnesium alloy forging temperatures can be selected. The following conclusions were made on the basis of the first stage of forging tests:

- Billet heating temperature equal to 350 °C is insufficiently low for the studied alloys;
- Billet heating temperature equal to 410 °C ensures improved plasticity of the alloys;
- The WE43 and AZ80 magnesium alloys are invalid for hammer forging in the designed technology;
- The AZ31 alloy manifests the best plasticity. The material did not crack in both the surveyed temperatures;
- The AZ61 alloy can be forged at a heating temperature of 410 °C. No cracking occurred (as opposed to the temperature of 350 °C);
- The formation of a forging lap constitutes a limitation for the application of the AZ31 and AZ61 alloys in the forging process. The problem becomes more severe as the height of the billet grows after upsetting, which confirms the simulations (Figure 14).

**Figure 15:**
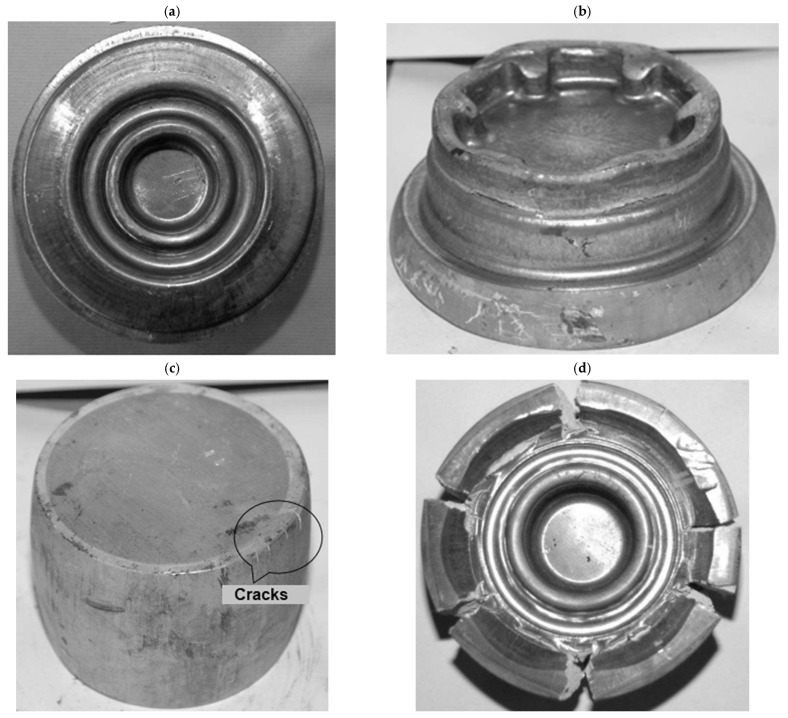
Results of industrial tests: (**a**) fault-free forging from the AZ31 alloy manufactured in the billet heating temperature of 420 °C, (**b**) peripheral cracking in the WE43 alloy forging heated to 450 °C, (**c**) radial cracking emerging during the upsetting of the AZ80 alloy heated to 450 °C, and (**d**) radial cracking of the forging from the AZ61 alloy heated to 450 °C (own study based on the literature [53,54]).

With the parameters outlined above, fault-free forgings were produced solely from the AZ31 alloy (Figure 15a). The WE43 alloy manifested significantly higher plasticity when heated to 450 °C than at 410 °C. Cracking occurred as a result of the initial forging and emerged peripherally (Figure 15b). On the other hand, for the AZ61 and AZ80 alloys, plasticity deteriorated with the growth of the temperature. As for the AZ80 alloy, peripheral cracking emerged at the edges of the billet in the first phase of upsetting (Figure 15c). The AZ61 alloy underwent complete disintegration (Figure 15d). For these cases, it can be inferred that hot brittleness occurred. Based on the results, a conclusion can be made that hammer forging enables fault-free products to be obtained exclusively from the AZ31 alloy. The remaining alloys require other forging conditions.

Unlike cast wheels, forged wheels are characterized by a fine-grained structure and better mechanical properties. The process of forging wheels allows a favorable grain pattern to be obtained and the alignment of the directional pattern along the spokes of the wheel to be optimized. Although forged magnesium wheels are 25 percent lighter than cast wheels, they are less commonly used due to their high production cost. The most frequent industrial application for forged magnesium wheels is in the automotive industry. An example of a magnesium wheel used in the automotive industry can be seen in a motorcycle racing wheel formed by manufacturer Marchesini using the forging method [9]. Another example might be a one-piece magnesium wheel for a racing car made by the forging method from a cast billet [48]. Since the formability of the cast billet was good, a process similar to reverse extrusion was adopted for forging the rim, and the disk was formed simultaneously during a single wheel-forming step. The spinning process, normally used to form the rim, was not necessary [48].

### 3.3. Extrusion of Magnesium Wheels in a Closed Die

**Figure 16:**
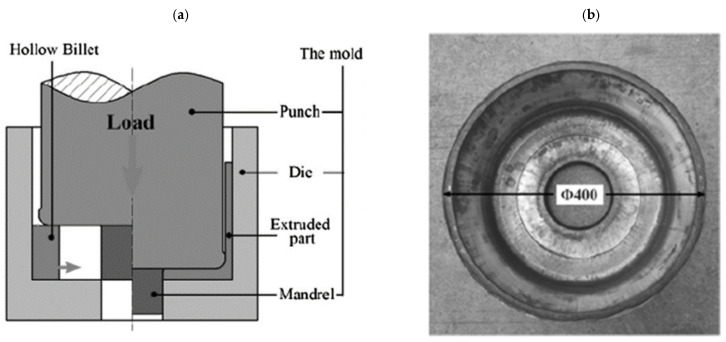
Diagram of the wheel extrusion process in a closed die (**a**) and the formed wheel forging by this technology (**b**) (own study based on the literature [54]).

The next method used for forming magnesium wheels is extrusion in a closed die (Figure 16) [20,33,55,56]. This technology produces wheels with straight geometry [57]. A cylindrical hollow billet prepared from the AZ80 magnesium alloy from a casting was used for the process. Subsequently, the billet was carried out at 380 °C. The developed technology and dies have many advantages, such as an improved filling of the flange, high production precision, and low surface roughness. The work of [55] also presents fatigue life tests for rim and disk samples cut from forgings shaped by the developed technology. The results of the number of cycles to failure as a function of the total strain amplitude for rim and disk cut samples were compared with the AZ80 magnesium alloy formed by rolling and extrusion. From the comparison, it was observed that the fatigue life of the alloy increased as the strain amplitude decreased. Improved fatigue resistance is observed for specimens cut from the rim rather than from the disk at the same strain amplitude. Similar fatigue performance was observed for specimens cut from the disk and rolled from the AZ80 magnesium alloy. The extruded AZ80 magnesium alloy exhibited a shorter and dissipated fatigue life.

**Figure 17:**
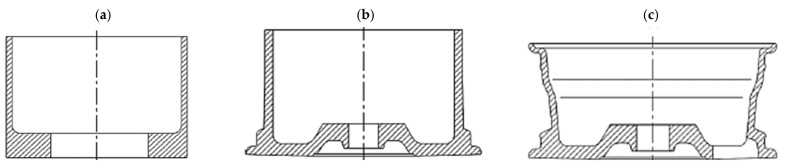
Diagram of the process of manufacturing magnesium wheels by extrusion: (**a**) extruding preform; (**b**) forging front lip; and (**c**) expanding rim and back lip (own study based on the literature [54]).

**Figure 18:**
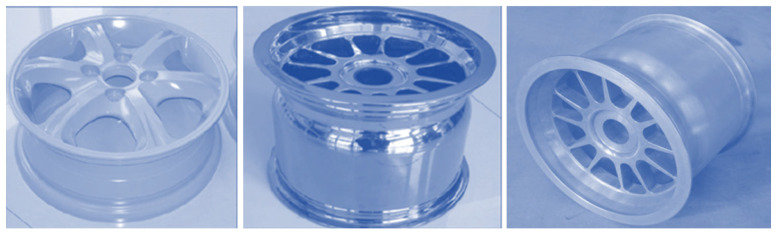
Magnesium wheel forgings obtained by extrusion method (own study based on the literature [53,54]).

Another example of the technological process of manufacturing a magnesium wheel by counter-rotating extrusion from a hollow billet was presented by the authors of paper [33]. The process diagram is shown in Figure 17. The concept of this technology is that the billet in the form of a hollow billet from the AZ80 magnesium alloy is placed in a closed die and then subjected to a backward extrusion process at a temperature ranging from 320 °C to 380 °C. The formed extruded preform is shown in Figure 18. The front lip of the wheel is then forged from the preform. The contour of the rim and the rear lip of the wheel are finally achieved by expansion. Examples of magnesium wheels formed by extrusion are shown in Figure 18.

The two presented examples [33,55] of manufacturing magnesium alloy wheels using the hollow billet and extrusion under isothermal conditions show that the forming load can be effectively reduced. Additionally, the strength of the spoke area can be improved. The produced AZ80 alloy rims, using the extrusion method, successfully passed the impact test, radial fatigue test, and bending fatigue test. The results from these tests showed that the formed rims can meet the requirements of the automotive industry and have already been implemented in many enterprises. Other authors [58,59,60] presented a similar process for the manufacture of the wheel, i.e., backward extrusion of a cylindrical billet by means of a set of circular dies. The process was conducted on a hydraulic press. The top and bottom dies were heated by a ceramic heating jacket, ensuring a suitable forming temperature. The billet was heated in a resistance furnace for 30 min. It was proposed to line the die and the preform with a carbon-based grease. The heated billet was placed in the bottom die. The final forging of the magnesium wheel is obtained when the top die reaches its final position, and the billet fills the space between the dies. The study used the AZ80A alloy. The billet used in the test was selected from a commercial alloy, homogenized at 420 °C for 16 h, and air-cooled [58,61]. A premise was made that the travel speed of the top die is 1 mm/s and the die temperature is 420 °C.

A similar schematic of the process and the magnesium wheel formed during backward extrusion was presented by the authors in paper [62]. The study was conducted for AZ80 ingots, which were homogenized at 420 °C for 24 h prior to forging. The backward extrusion test was conducted in isothermal conditions at 420 °C. The process was executed on an 800 kN forging press. The top die travel speed was 3 mm/s. In order to reduce friction between the billet and the dies, oil-based graphite was applied. The size of the formed wheel was 6 inches. 

### 3.4. Hybrid Methods for Forming Magnesium Wheels

#### 3.4.1. Casting and Die Forging

**Figure 19:**
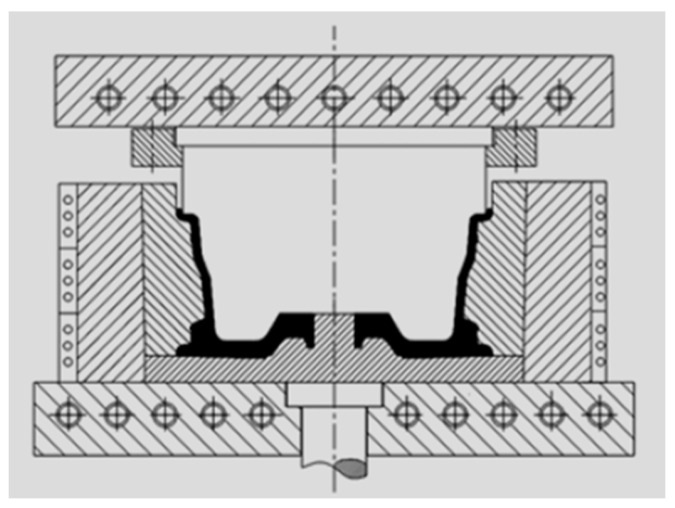
Diagram of the die device for magnesium wheel forging (own study based on the literature [54]).

In the paper [54,62], the authors presented a new process for forming a magnesium wheel using the precision die-forging method with a combined bottom die design. The forging technology with a modular bottom die conceptually resembles the backward extrusion technology. The structure of the bottom die meets the molding requirements and allows the material to be easily removed from the die. The die is not monolithic due to the geometry of the wheel. Otherwise, the material could not be removed from the die after forging. Therefore, the bottom die is divided into two parts. In this process, the wheel forging is formed from a cast billet. The AZ80 alloy for the wheel was produced by the semi-continuous direct-casting method. The billets were machined from the ingot, homogenized at 400 °C for 12 h, and cooled in air. The machined casting was subjected to upsetting before forging. For magnesium alloys, it was recommended that the process take place under isothermal conditions. For this purpose, the tool system was heated by electric heating elements built into the top plate, punch, base, and die. Insulating layers were also used to prevent heat transfer from these elements to the tool frame and press. A thermocouple was placed in the die to monitor the temperature of the die. The special heating systems (marked with circles) allowed the maintenance of the temperature of the tools close to that of the material being deformed during the relatively slow process. As a result, the formed rim of the wheel with a small thickness did not cool down from the tools and retained the appropriate plastic properties. A diagram of the process is shown in Figure 19. The billet for forging was heated to temperatures of 360–400 °C. The forging process was carried out on a 6.3 MN hydraulic press with a tool movement speed of 16 mm/s and with colloidal graphite lubrication. After the forging process, the lower die extends to remove the wheel forging. The study showed that the tensile strength of the samples cut from the obtained product was 300 ÷ 320 MPa, and elongation exceeded 10%. Such a wheel-forging technology and the developed dies have numerous advantages, such as better flange filling, high production precision, low surface roughness, and convenient die unloading for forming a forging with a geometry close to the final wheel.

#### 3.4.2. Die Forging and Flow-Forming Process

**Figure 20:**
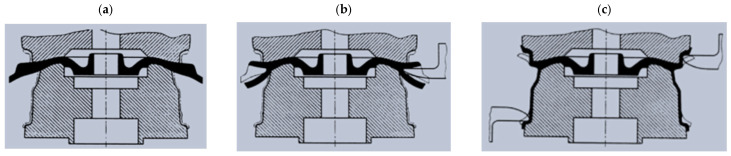
Diagram of the flow-forming processes of magnesium wheel: (**a**) initial position; (**b**) splitting flange; (**c**) flow-forming the rim and calibrating the rim contour (own study based on the literature [54]).

Forged magnesium wheels are produced by various techniques. Among them, it is possible to distinguish the method of forming forged Mg alloy wheels in two operations consisting of die forging and flow forming [33,54]. In the first operation, the wheel disk is forged in the impression, while in the second operation, the wheel rim is flow-formed. The billet used for this technological process is in the form of castings after homogenization. Prior to the forging and flow-forming process, the billet is heated to the appropriate temperature to ensure the required formability of magnesium alloys. The metal forming of magnesium alloy wheels is a difficult task due to their narrow range of forming temperature parameters and sensitivity-to-strain rate. Therefore, the plastic-forming process of magnesium wheels is carried out hot on forging machines with low operating speeds while maintaining isothermal temperature conditions during deformation. The forging process consists of three stages on a hydraulic press with different pressing powers. The first forging operation with a high reduction rate produces a semi-finished wheel shape. The final shape of the wheel disk is mainly obtained by the second forging. The forming process is completed with a deburring operation. The flow-forming operation proceeds in three steps: splitting the forged flange, flow-forming the rim, and calibrating the rim contour (Figure 20). Based on the proposed technology, a magnesium alloy ZK30 rim was produced. The wheel manufactured in this way has lower mass (by 35%) than that of the wheel produced from an aluminum alloy. The authors of the article argue that the technology ensures a favorable flow direction, which improves the strength properties of the wheel. In spite of that, fatigue tests indicated that the durability of the wheel (measured by the number of load cycles) amounts to merely 8.5% of the aluminum wheel’s durability (the specific aluminum alloy was not disclosed). In order to improve durability, the thickness of the wheel’s walls ought to be increased. 

A similar solution for forming magnesium wheels through a complex process of forging and flow forming is presented in the patent [49]. The billet, in the form of a cylinder of cast magnesium alloy heated to the temperature ranging from 250 °C to 450 °C, is forged in two die-forging operations by means of dies heated to 250 °C and 450 °C and a processing speed ranging from 2 mm/s to 13 mm/s. It is flow-formed from the semi-finished product heated from 150 °C to 400 °C in the third operation. The final shape of the magnesium alloy vehicle wheel is obtained by machining the forging.

Advantages of this method consisting of die forging and flow forming include material economy, high production efficiency, low processing overheads, and high strength [33,49,54].

#### 3.4.3. Die Forging and Spin Forging Process

**Figure 21:**
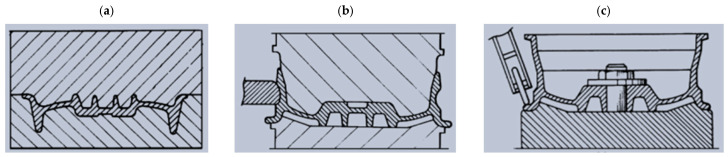
Diagram of the complex technological process of forming magnesium wheel by die forging and spin forming: (**a**) die forging of the billet; (**b**) spin forming; and (**c**) roll forming (own study based on the literature [54]).

Forged magnesium wheels are also produced by technological processes consisting of die forging and spin forming [33,54]. This technology consists of three processing steps: die forging, rim spinning, and then rim edge-roll forming (Figure 21). The billet is prepared by casting and then forged to obtain a wheel impression with a shape similar to that of the final product. A car wheel is obtained by forging from a billet using a forging machine with upper and bottom dies. The wheel forging is then subjected to solution and heat treatment. After the treatment is completed, spin forging is carried out. While the wheel is rotated with the mandrel, the roll is pressed into the wheel rim so that the rim can be finished by spin machining. Ultimately, the wheel is treated with a shaping roller to improve its corrosion resistance. It is pressed to the edge part of the rim while the rotating platform, together with the wheel, rotates.

A similar solution is presented in the patent [63] and publication [64]. The authors propose that the process includes:

- Heating, in which a cylindrical magnesium alloy billet is heated to the forging temperature;
- Pre-forging, in which the initial forming of the spokes and rim occurs by backward extrusion. However, the outer rim is not formed;
- Final forging, in which the outer rim is finally formed, and the final formation of the rim section is carried out by forward extrusion;
- Inward spin-forming to complete the enlargement of the wheel rim and the spinning flange-forming of the inner rim.

Although the combined process of die forging and spinning can significantly reduce the tonnage of forming equipment, its efficiency is lower than the single-step backward extrusion process.

### 3.5. Flow Control-Forming of magnesium wheels

**Figure 22:**
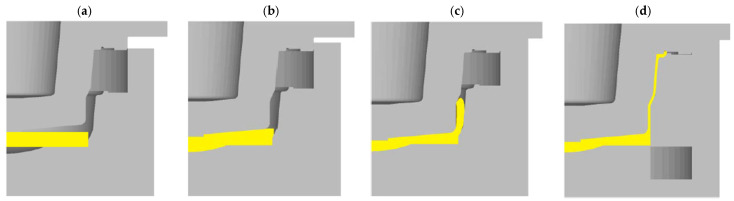
Diagram of the process of forming magnesium wheels by the method of flow control-forming: (**a**) cylindrical compression; (**b**) backward extrusion; (**c**) extrusion-shearing; and (**d**) closed-die forging [6].

In the work of [65], the authors presented a new alternative technology for forming magnesium wheels using the flow control-forming (FCF) method. The FCF method consists of two stages: the forming of the rim and the forming of the spokes. Due to the stress characteristics of wheel forming, a relatively simple process can be distinguished in the FCF method, such as:

- Cylindrical compression (Figure 22a);
- Backward extrusion (Figure 22b);
- Extrusion-shearing (Figure 22c);
- Closed-die forging (Figure 22d).

**Figure 23:**
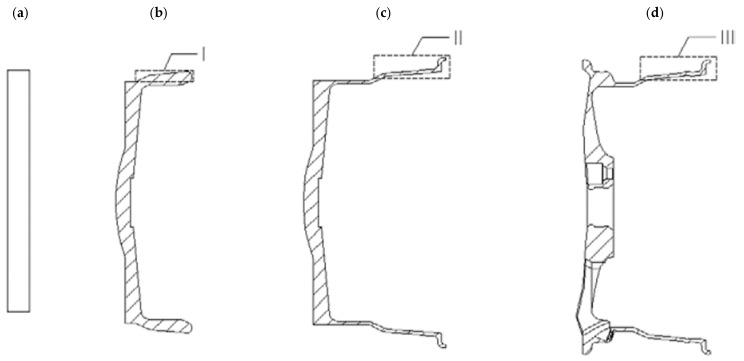
Diagram of the change in the product shape in the different stages of the flow control-forming process: (**a**) billet; (**b**) preforming; (**c**) forming the rim; and (**d**) forming spokes [65].

The examination of flow control-forming was done at 415 °C in isothermal conditions and with a constant friction factor of 0.21. Simulations for various extrusion speeds (5, 10, 15, and 20 mm/s) were made. The most important aspect of the flow control-forming method is controlling the direction of the metal flow and the forming sequence. In the process of forming a magnesium wheel, the flow of the wheel rim material is first controlled and then forced to flow to a certain position (Figure 23b). Then, the billet is further extruded into the final shape of the rim (Figure 23c). In Figure 23, the change in the shape of the billet can be traced through various stages of the flow control-forming process.

**Figure 24:**
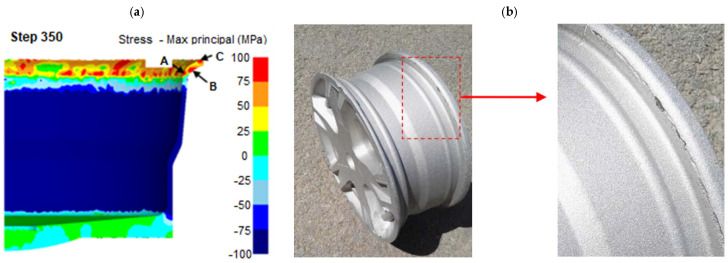
Cracking emerging in the flow control-forming process of the AZ80 magnesium wheel was established in the course of (**a**) FEA simulations and (**b**) experimental tests [65].

The main objective of the study of the flow control-forming process was the analysis of the deformation force and deformability of the AZ80 magnesium wheel. The results of the study revealed that the maximum deformation force was below 2200 t throughout the process and varied insignificantly for the surveyed extrusion speeds. Based on the study, a conclusion was made that the main limitations of the flow control-forming process encompass cracking. The limitations were verified during tests of the surveyed magnesium wheel (Figure 24). The results of the work [65] confirmed that the flow control-forming method employed for the manufacture of magnesium wheels decreases the deformation force. The improvement in deformability of the magnesium wheel rim and the impact of extrusion-shearing on the wheel require further studies. 

### 3.6. Cold Spray Technology

Recently, cold spray technology emerged as a potential tool to repair metal parts, and it takes a few minutes. The technology can be used to quickly repair magnesium wheels [66,67]. The process involves particles of a selected grade of alloy being mixed with nitrogen and helium and gradually applied to the damaged wheel to restore the desired surface. A robot controls the movement of the sprayer. This method is also used to apply anti-corrosion coatings to magnesium wheels produced by other manufacturing techniques like casting, forming, and machining [16,68]. 

There are two types of cold spraying, i.e., high-pressure spraying and low-pressure spraying. For high-pressure cold spraying, a working gas in the form of nitrogen or helium is used at a pressure of more than 1.5 MPa, with a flow rate of more than 2 m^3^/min and a heating power of 18 kW. This type of spraying is used for spraying pure powders [69] of metals and their alloys with a size of 5–50 µm. For low-pressure cold spraying, the working gas is used in the form of compressed gas with a pressure of 0.5 to 1 MPa, a flow rate of 0.5 to 22 m^3^/min, and a heating power of 3 to 5 kW. This type of spraying is used for spraying a mechanical mixture of metal and ceramic powders. The inclusion of a ceramic component in the mix provides high-quality coatings with a relatively low energy consumption. 

Cold spraying has many advantages that make this technology potentially very competitive. Being a cold process, the initial properties of the particles are preserved, and heating of the substrate is minimal, resulting in a microstructure of cold-treated coatings in which there is no income to melt and solidify. In addition, the technology makes it possible to spray thermally sensitive materials and very different combinations of materials since the adhesion mechanism is purely mechanical. Other significant advantages of cold spraying include the following: high thermal and electrical conductivity of coatings; high density and hardness of coatings; high homogeneity of coatings; no melting; ability to spray fine particles (5–10 µm), nanomaterials and amorphous materials; minimal surface preparation; low energy consumption; ability to achieve complex shapes and internal surfaces; high productivity due to a high power factor; high deposition rates and efficiency; environmentally friendly because there is no toxic waste; and increased work safety due to the absence of high-temperature gas and radiation streams. 

As for limitations in the application of this method, for example, it is difficult to spray hard and brittle materials because in this case, mechanical adhesion through plastic deformation may not be as effective as with plastic particles. Other problems may include near-zero plasticity in the sprayed state, the need for a plastic substrate, difficulties in processing pure ceramics and some alloys as curing alloys, the high cost of helium, contamination, and erosion of the nozzle.

### 3.7. Sustainable Development in Terms of Magnesium Wheel Production 

In recent years, when developing new technologies for the manufacturing of magnesium wheels, closer attention has been paid to the adherence to sustainable development principles. This is especially valid for environmental protection, where there is a tendency to improve or replace energy-intensive, material- and labor-consuming magnesium wheel-manufacturing methods with optimized processes. Producers of Mg wheels and research centers are increasingly interested in low- and zero-waste technologies and those limiting the number of manufacturing operations to a bare minimum. This enhances the use of energy and materials and minimizes the impact of businesses on the natural environment by reducing the volume of waste to be recycled and utilized. In order to increase productivity and reduce costs in magnesium wheel production, Dewei Technology and Dingxin Magnesium have built a production line for a one-step forming process, in which the billet is heated once, and only one set of dies is used to form the wheel forging [70]. Other promising magnesium wheel-forming technologies include backward extrusion, which is low-waste and enables the energy required to manufacture the semi-finished product to be reduced. Further prospective technologies, in terms of the quality of the product and environmental friendliness, encompass hybrid methods combining various manufacturing technologies, some of which enable a virtually finished product to be obtained and the volume of waste to be reduced. Such precise and efficient technologies enable a global tendency for sustainable production and consumption to be promoted in order to break the link between economic growth and the degradation of the environment. 

Due to the fact that magnesium is a recyclable metal, it is possible to recycle magnesium wheels. The recycling of magnesium alloys requires merely 5% of the energy needed to manufacture the original alloy. The industry actively promotes recycling due to its positive environmental impact. Life cycle studies concerning greenhouse gasses, such as Prentice (CSIRO) and Ehrenberger (DLR), take into consideration the recycling of material after the life cycle has been completed in order to obtain the full perspective of the environmental benefits emerging from the application of magnesium throughout the life cycle. For economic reasons, the processing of end-of-life magnesium wheels is usually achieved by shredding. Once the wheels are shredded, different grades of magnesium alloys and their integrated components are mixed. Shredded magnesium can be contaminated with iron, nickel, and copper from coatings and fasteners, all of which have a detrimental effect on the metal’s corrosion resistance. Re-melting alloys consumes up to 50% of the energy required for distillation. The same quality criteria, in terms of chemical composition and oxide content, must be met for both recycled alloy ingots and virgin metal.

Scrap, in the form of technological waste generated in the production of magnesium wheels, is also sent for recycling. During the production of wheels by casting methods, waste is generated in the form of elements of the casting system and the geometry of the supply. In the production of magnesium wheels by metal-forming methods, technological waste is produced in the form of flashings. On the other hand, in the technology of manufacturing magnesium wheels by machining methods, chips are produced. Re-melting magnesium chips, due to magnesium’s susceptibility to oxidation, involves greater alloy losses than in the case of solid scrap. The chips require washing with water to remove machining lubricants. An alternative to melting the chips is to press them into briquettes, which allows the chips to be used as an alloying material for aluminum. Magnesium briquettes can be directly hot-extruded as part of the solid recycling method since the metal does not melt and no special protective environment is required. A study conducted at Harbin University of Science and Technology in China has shown that solid-state recycling of magnesium alloy chips is an efficient recycling method [71,72].

Researchers Tzamtzis S. and another from the Brunel Center for Advanced Solidification Technology, in an article [73], presented the melt-conditioned high-pressure die casting (MC-HPDC) process they developed for recycling magnesium alloy cast components. The MC-HPDC process involves subjecting an alloy to intense shear before it is poured into a metal die. Researchers have shown that the developed melt-conditioned high-pressure die-casting process, compared to conventional high-pressure die-casting, makes it possible to shape castings with a fine and uniform microstructure and thus have better mechanical properties.

Researchers Dechner F. et al., from the Magnesium Innovation Centre MagIC, Institute of Materials Research, Helmholtz-Zentrum Geesthacht, Max-Planck-Strae 1, 21502 Geesthacht, Germany, in an article [74], presented ongoing research solving the problem associated with mixed alloy elements and impurities during recycling within a single alloy system. Six alloys were developed by high-pressure casting to prepare a secondary magnesium alloy system for mixed post-consumer scrap from end-of-life vehicles. Secondary alloys are being prepared to create new components from melted scrap with a minimum amount of primary metal while achieving the desired and needed alloy composition. This research promotes the recycling of end-of-life vehicles through re-alloying that uses a minimum amount of primary metal and energy.

Despite the various existing methods of recycling magnesium wheels, there is still a possibility for improvement. Further development of recycling methods is becoming a key area for future research. 

## 4. Summary

Magnesium alloys, classified as lightweight metals, are a group of forward-looking engineering materials. Their low weight and good mechanical properties have led to considerable interest in the engineering and transportation equipment industries concerning the manufacture of components from these materials. The continuous reduction in structural weight has been one of the main production priorities in various industries in recent years. As a result, magnesium alloy components are increasingly used in mechanical engineering. Primary customers for magnesium parts are the automotive and aerospace industries, where reducing the weight of structures can directly translate into improved transportation dynamics. The article reviews and systematizes the available knowledge in the field of magnesium wheels in terms of construction, their application in various industries, and production methods. 

The analysis of magnesium wheels in terms of construction enabled wheels of different geometries to be distinguished, such as one-piece or with complex geometry (three-piece or five-piece wheels). Five-piece magnesium wheels are the most complex and costly but have high quality and strength. In terms of design, magnesium wheels can have different shapes, including multi-spoke, with holes, or with frames. The shape of magnesium wheels will change in the future and will depend on the styling trends of the time. 

An analysis of magnesium wheels by industrial applications has resulted in wheels for cars, motorcycles, bicycles, and wheelchairs being distinguished.

The literature review on magnesium wheel production methods enabled the analysis of technologies currently used for producing magnesium alloy wheels to be conducted. It also allowed possible directions for further development to be indicated. Prospective directions in the development of methods for producing magnesium wheels include hybrid methods combining various manufacturing processes, cold spray technology, and research on recycling magnesium wheels.

A detailed review of the types of magnesium wheels currently used in the industry was also conducted for the ForMag project, which aims to develop a new, efficient technology for forming magnesium alloy wheels for light vehicles from die-cast preforms. The compiled knowledge of the types of magnesium wheels used in the industry and their applications will enable the selection of magnesium wheel geometries for the ForMag project and the design of new hybrid technologies based on casting and die forging. The development of hybrid technology based on casting and die forging makes possible a reduction in the number of operations needed to obtain a wheel compared to multi-stage forming. It makes it possible to reduce process waste as a result of using a shaping billet optimized for the part. The use of a cast billet for the forging process makes it possible to form hard-to-deform magnesium alloys due to the fact that they have not been plastically processed and that their deformability is better.

## Figures and Tables

**Table 1:** Comparison of magnesium wheels with aluminum and steel wheels (own study based on the literature [3,5,6]).

	Magnesium Wheels	Aluminum Wheels	Steel Wheels
Advantages	Lighter than steel and aluminum wheels. Greater fuel efficiency, better braking, and longer tire life. Magnesium wheels have much better heat transmission than steel wheels. Better heat transfer helps dissipate heat from the brakes and reduces the risk of brake failures, which often result from overheating. Magnesium wheels have damping properties that exceed 50 times that of aluminum wheels. They can significantly reduce the vehicle’s vibration load, especially in components such as the engine, suspension, and transmission. This effect contributes to improving the overall performance of the vehicle and extends its life.	Lighter than steel wheels. Better fuel economy, better braking, and better tire life. Aluminum wheels have much better heat transmission than steel wheels. Better heat transfer helps dissipate heat from the brakes and reduces the risk of brake failures, which often result from overheating.	Steel wheels cost less than aluminum and magnesium wheels. Steel wheels are heavier than aluminum and magnesium wheels, which can be advantageous when driving in winter. Steel wheels provide the strength and durability required by trucks and heavy equipment.
Disadvantages	Magnesium wheels are susceptible to corrosion. Therefore, protective coatings are applied to them. Magnesium wheels are more expensive than aluminum and steel wheels, which is why they are used for exclusive cars and racing cars.	Less hard and durable than steel wheels. More prone to potential damage than steel wheels. More expensive to purchase and repair than steel wheels. Steel wheels that bend can be repaired, but aluminum wheels do not bend; rather, they break and need to be replaced with newer ones. Aluminum wheels, compared to steel wheels, are susceptible to galvanic corrosion. This causes air leakage from tires if preventive measures are not taken.	They are heavier than magnesium aluminum wheels and can, therefore, be less fuel-efficient, have longer braking distances, and a shorter lifetime.

**Table 2:** Wheel optimization [12].

Name	Wheel (a)	Wheel (b)	Wheel (c)	Wheel (d)	Wheel (e)	Wheel (f)
Size, L (mm)	0	5	7.5	10	12.5	15

**Table 3:** Results of static structural analysis for 5-twin-spoke and multi-spoke wheel models made of aluminum 6061 and magnesium AZ91D alloys [23].

Material	Maximum Total Deformation (mm)		Maximum von Mises Stress (MPa)	
	5-Twin-Spoke	Multi-Spoke	5-Twin-Spoke	Multi-Spoke
Aluminum 6061 alloy	0.55174	0.34889	41.048	35.784
Magnesium AZ91D alloy	0.84468	0.53375	41.077	35.681

**Table 4:** Comparison of magnesium wheels with aluminum and steel wheels (own study based on the literature [6,47]).

	Low-Pressure Die Casting	High-Pressure Die Casting
Advantages	- Eight to ten times cheaper technology than high-pressure die casting; - Less wear on the metal mold than in high-pressure die casting; - Forming castings are less porous due to the prevention of contact between molten metal and air.	- Lower technology costs due to high productivity; - It is applicable to parts with complex geometries; - Cold chamber process can be used for larger parts; - For small and thin-walled parts, a hot chamber process can be used.
Disadvantages	- Technology is labor intensive and needs trained operators	- Less lifetime and pressure tightening resistance; - High porosity of castings.

## Data Availability

Data is contained within the article.

## References

[1] Song J., Chen J., Xiong X., Peng X., Chen D., Pan F. (2022). Research advances of magnesium and magnesium alloys worldwide in 2021. J. Magnes. Alloy..

[2] Kumar D.S., Sasanka C.T., Ravindra K., Suman K.N.S. (2015). Magnesium and Its Alloys in Automotive Applications—A Review. Am. J. Mater. Sci. Technol..

[3] Kulekci M. (2008). Magnesium and its alloys applications in automotive industry. Int. J. Adv. Manuf. Technol..

[4] Dziubinska A. (2023). Investigation of a New Screw Press Forming Process for Manufacturing Connectors from ZK60 Magnesium Alloy Preforms. Materials.

[5] Zhang Z., Zhang Y., Zhang L., Pan A., Liu J. (2016). Application of micro-arc oxidation technology in die magnesium alloy wheels in mass production. Key Eng. Mater..

[6] Wang M. (2023). An Industrial Perspective on Magnesium Alloy Wheels: A Process and Material Design. Mater. Sci. Appl..

[7] Leister G. (2018). Wheels. Passenger Car Tires and Wheels.

[8] Burk G. (2018). Porsche 911 Turbo Carbon Wheel, 9th International Munich Chassis Symposium 2018.

[9] Agnew S.R. (2004). Wrought magnesium: A 21st century outlook. JOM.

[10] https://www.formula1-dictionary.net/wheels.html.

[11] Jiang X., Lan X. (2022). Magnesium Alloy Wheel Structure Design and Wheel Casting Process Performance Analysis. J. Mater. Sci. Eng. B.

[12] Jiang X., Liu H., Lyu R., Fukushima Y., Kawada N., Zhang Z., Ju D. (2019). Optimization of Magnesium Alloy Wheel Dynamic Impact Performance. Adv. Mater. Sci. Eng..

[13] Eliezer D., Aghion E., Froes F.H. (1998). Magnesium science, technology and applications. Adv. Perform. Mater..

[14] Qi Y., Wang H., Chen L., Zhang H., Chen G., Chen L., Du Z. (2020). Preparation and mechanical properties of ZK61-Y magnesium alloy wheel hub via liquid forging—Isothermal forging process. Metals.

[15] Li Z., Luo A.A., Wang Q., Peng L., Zhang P. (2016). Fatigue Properties of Cast Magnesium Wheels. Metall. Mater. Trans. A Phys. Metall. Mater. Sci..

[16] Villafuerte J., Zheng W. (2007). Corrosion Protection of Magnesium Alloys by Cold Spray. Adv. Mater. Process. Mag..

[17] Liu B., Yang J., Zhang X., Yang Q., Zhang J., Li X. (2023). Development and application of magnesium alloy parts for automotive OEMs: A review. J. Magnes. Alloy..

[18] Karuppusamy S., Karthikeyan G., Dinesh S., Rajkumar T., Vijayan V., Basha J.K. (2016). Design and Analysis of Automotive Wheel Rim by using ANSYS and MSC Fatigue Software. Asian J. Res. Soc. Sci. Humanit..

[19] Theja S. (2013). Structural and Fatigue Analysis of Two Wheeler Lighter Weight Alloy Wheel. IOSR J. Mech. Civ. Eng..

[20] Siemionek E., Majerski K., Surdacki P., Novakova-Marcincinova E. (2023). Review of Magnesium Alloys Used in the Manufacture of Wheels for Light Vehicles. Adv. Sci. Technol. Res. J..

[21] Dietsche K.-H., Reif K. (2018). Automotive Handbook.

[22] Gebrehiwet L., Negussie Y., Tadesse A. (2022). Business Opportunity of Wheel Rim Manufacturing in Ethiopia. Int. J. Adv. Eng. Manag..

[23] Karim H., Ku P.X. (2022). Effect of design and material on structural rigidity of automobile wheel—An optimisation approach. J. Phys. Conf. Ser..

[24] Papenberg N.P., Gneiger S., Weißensteiner I., Uggowitzer P.J., Pogatscher S. (2020). Mg-alloys for forging applications—A review. Materials.

[25] Blawert C., Hort N., Kainer K.U. (2004). Automotive applications of magnesium and its alloys. Trans. Indian Inst. Met..

[26] Khatkar S.K. (2023). Hybrid magnesium matrix composites: A review of reinforcement philosophies, mechanical and tribological characteristics. Rev. Adv. Mater. Sci..

[27] Śliwa R.E., Balawender T., Hadasik E., Kuc D., Gontarz A., Korbel A., Bochniak W. (2017). Metal forming of lightweight magnesium alloys for aviation applications. Arch. Metall. Mater..

[28] Yang H., Huang L., Zhan M. (2010). Coupled thermo-mechanical FE simulation of the hot splitting spinning process of magnesium alloy AZ31. Comput. Mater. Sci..

[29] Höche D., Weber W.E., Gazenbiller E., Gavras S., Hort N., Dieringa H. (2021). Novel Magnesium Based Materials: Are They Reliable Drone Construction Materials? A Mini Review. Front. Mater..

[30] Gupta M., Gupta N. (2017). The Promise of Magnesium Based Materials in Aerospace Sector History and Current Trends of Magnesium in Aerospace Sector. Int. J. Aeronaut. Sci. Aerosp. Res..

[31] Kainer K.U. (2003). Magnesium—Alloys and Technology.

[32] Pan B.S., Gong H.L., Peng T.H., Zhang M. (2009). Magnesium Alloy Material Substitution Redesign Method for Wheels of Electric Bicycle. Adv. Mater. Res..

[33] Wang Q., Zhang Z.M., Zhang X., Li G.J. (2010). New extrusion process of Mg alloy automobile wheels. Trans. Nonferrous Met. Soc. China Engl. Ed..

[34] Czerwinski F. (2008). Magnesium Injection Molding.

[35] Dobrzanski L.A., Tanski T., Cizek L., Domagala J. (2008). Mechanical properties and wear resistance of magnesium casting alloys. J. Achiev. Mater. Manuf. Eng..

[36] Krajewski P., Verma R. (2014). Cast Magnesium Alloy Wheels. U.S. Patent.

[37] Luo A.A., Sachdev A.K., Apelian D. (2022). Alloy development and process innovations for light metals casting. J. Mater. Process. Technol..

[38] Yang Y., Ma C., Luo T., Ji G., Ge S., Shi B. (2014). A Magnesium Alloy Vehicle Wheel Hub Casting One Spinning Composite Forming Method. China Patent.

[39] Zhang Z., Wang Q., Li B., Zhang X., Li G. (2006). Casting Extruding Compound Shaping Method of Magnesium Alloy Automobile Hub. China Patent.

[40] Yang Q., Yang Y., Li H., Yu K. (2022). Extrusion Casting Forming Die for Magnesium Alloy Hub. China Patent.

[41] Luo A.A. (2013). Magnesium casting technology for structural applications. J. Magnes. Alloy..

[42] Zhang Z., Zhang Y., Zhang H., Pan A., Liu J. (2019). Research on Die Casting Process of Magnesium Alloy Motorcycle Wheel Based on New Engineering Construction. J. Phys. Conf. Ser..

[43] Wang Y.C., Li D.Y., Peng Y.H., Zeng X.Q. (2007). Numerical simulation of low pressure die casting of magnesium wheel. Int. J. Adv. Manuf. Technol..

[44] Zhang C., Fu Y., Wang H., Hao H. (2018). Multi-objective optimization of process parameters during low-pressure die casting of AZ91D magnesium alloy wheel castings. China Foundry.

[45] Ying F., Tang H., Peng T. Numerical simulation of low pressure die-cast of magnesium alloy wheel. Proceedings of the 2008 Asia Simulation Conference—7th International Conference on System Simulation and Scientific Computing, ICSC 2008.

[46] Guo W., Ma Z., Zhang P., Shu Z., Ye S., Chen Z. (2009). Low-Pressure Die Casting Machine for Magnesium Alloy Automobile Wheels. China Patent.

[47] Mathaudhu S.N., Luo A.A., Neelameggham N.R., Nyberg E.A., Sillekens W.H. (2014). Advances in Manufacturing Processes for Magnesium Alloys. Essential Readings in Magnesium Technology.

[48] Fujita M., Sakate N., Hirahara S., Yamamoto Y. (1995). Development of magnesium forged wheel. SAE Tech. Pap..

[49] Cui K., Ren P., Tian Y., Wang Z., Wang T., Liu T. (2014). Magnesium Alloy Wheel Forging-Spinning Composite Forming Method. China Patent.

[50] Liu Z., Li J., Liu X., Li J., Wu D. (2011). Magnesium Alloy Steering Wheel. China Patent.

[51] Xiong X. (2021). Production Method of High-Strength Light-Weight Forged Magnesium Alloy Hub. China Patent.

[52] Tong Z. (2018). Inverted Extrusion Die Forging Method Used for Magnesium Alloy Hub. China Patent.

[53] Dziubińska A., Gontarz A., Dziubiński M., Barszcz M. (2016). the Forming of Magnesium Alloy Forgings for Aircraft and Automotive Applications. Adv. Sci. Technol. Res. J..

[54] Gontarz A. (2016). Die Forging of Magnesium Alloys.

[55] Zhao X., Gao P., Zhang Z., Wang Q., Yan F. (2020). Fatigue characteristics of the extruded AZ80 automotive wheel. Int. J. Fatigue.

[56] Zhao X., Gao P., Chen G., Wei J., Zhu Z., Yan F., Zhang Z., Wang Q. (2021). Effects of aging treatments on low-cycle fatigue behavior of extruded AZ80 for automobile wheel disks. Mater. Sci. Eng. A.

[57] Liu X. (2017). A Kind of Magnesium Alloy Auto Hub Forging Method. China Patent.

[58] Jiang Y., Zhu Y., Le Q., Liao Q., Yin Z., Zhang X., Wang P. (2024). Finite element simulation and industrial validation for DRX evolution of magnesium alloy thin-walled wheel formed by backward extrusion 1 Highlights. Thin-Walled Struct..

[59] Jiang Y., Le Q., Liao Q., Hu C., Guo R., Yu X., Hu W. (2023). Simulation research on the rotating back extrusion process for magnesium alloy wheel. Int. J. Mater. Form..

[60] Jiang Y., Zhu Y., Le Q., Liao Q., Zhou W., Wang P., Wang T. (2022). Effect of truncated cone billet on single-step back extrusion forming process of magnesium alloy wheel. J. Mater. Res. Technol..

[61] Jiang Y., Ren L., Le Q., Liao Q., Zhu Y., Zhou W., Zhao T. (2023). Numerical simulation of billet height-diameter ratio on magnesium alloy automobile wheel formed by back extrusion. Int. J. Adv. Manuf. Technol..

[62] Wang Q., Zhang Z.M., Zhang X., Yu J.M. (2008). Precision forging technologies for magnesium alloy bracket and wheel. Trans. Nonferrous Met. Soc. China Engl. Ed..

[63] Sun H., Li J., Liu X. (2015). Magnesium Alloy Wheel Forging Forming Method. China Patent.

[64] Zhang Z., Yu J., Xue Y., Dong B., Zhao X., Wang Q. (2023). Recent research and development on forming for large magnesium alloy components with high mechanical properties. J. Magnes. Alloy..

[65] Zhao M.J., Wu Z.L., Chen Z.R., Huang X.B. (2017). Analysis on flow control forming of magnesium alloy wheel. IOP Conf. Ser. Mater. Sci. Eng..

[66] Ashokkumar M., Thirumalaikumarasamy D., Sonar T., Deepak S., Vignesh P., Anbarasu M. (2022). An overview of cold spray coating in additive manufacturing, component repairing and other engineering applications. J. Mech. Behav. Mater..

[67] Champagne V.K., Helfritch D., Leyman P.F. (2008). Magnesium repair by cold spray. Plating and Surface Finishing.

[68] Chakradhar R.P.S., Chandra Mouli G., Barshilia H., Srivastava M. (2021). Improved Corrosion Protection of Magnesium Alloys AZ31B and AZ91 by Cold-Sprayed Aluminum Coatings. J. Therm. Spray Technol..

[69] Sofia D., Macrì D., Barletta D., Lettieri P., Poletto M. (2021). Use of titania powders in the laser sintering process: Link between process conditions and product mechanical properties. Powder Technol..

[70] Jiang Y., Le Q., Zhu Y., Liao Q., Wang T., Bao L., Wang P. (2024). Review on forming process of magnesium alloy characteristic forgings. J. Alloys Compd..

[71] Wu S., Ji Z., Hu M., Huang Z., Tian C., Wu M. (2018). Microstructure and Mechanical Properties of AZ31B Magnesium Alloy Prepared by Solid State Recycling. Xiyou Jinshu Cailiao Yu Gongcheng/Rare Met. Mater. Eng..

[72] Xu H., Ji Z., Hu M. Semi-solid microstructure of AZ91D magnesium alloy recycled from waste chips during heat treatment. Proceedings of the 2012 7th International Forum on Strategic Technology, IFOST 2012.

[73] Tzamtzis S., Zhang H., Xia M., Babu N.H., Fan Z. (2011). Recycling of high grade die casting AM series magnesium scrap with the melt conditioned high pressure die casting (MC-HPDC) process. Mater. Sci. Eng. A.

[74] Fechner D., Blawert C., Hort N., Dieringa H., Kainer K.U. (2013). Development of a magnesium secondary alloy system for mixed magnesium post-consumer scrap. Mater. Sci. Eng. A.

